# Supplementary material for: Sequential and coordinated control of human plasma cell differentiation by IRF4 and BLIMP1 utilizing a discriminating ISRE/EICE motif lexicon
Source: bioRxiv. 2026 Jun 19:2026.06.15.732353. Preprint. [Version 1] doi: 10.64898/2026.06.15.732353 (PMC13307996; doi:10.64898/2026.06.15.732353)
Supplement: Supplement 1 [file NIHPP2026.06.15.732353v1-supplement-1.pdf]

bioRxiv preprint doi: https://doi.org/10.64898/2026.06.15.732353; this version posted June 19, 2026. The copyright holder for this preprint (which was not certified by peer review) is the author/funder, who has granted bioRxiv a license to display the preprint in perpetuity. It is made available under a CC-BY 4.0 International license.

Fig. S1

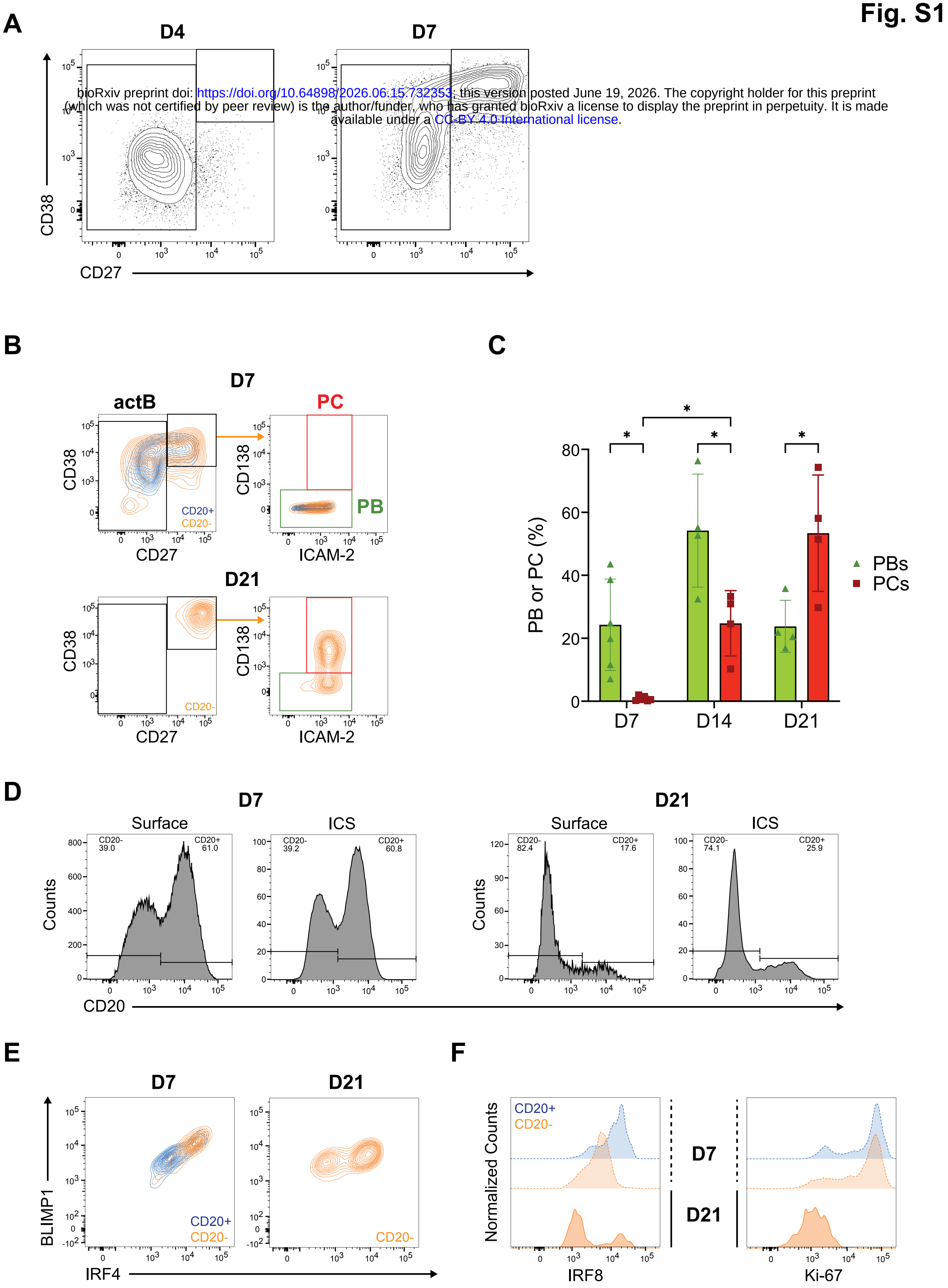

bioRxiv preprint doi: https://doi.org/10.64898/2026.06.15.732353; this version posted June 19, 2026. The copyright holder for this preprint (which was not certified by peer review) is the author/funder, who has granted bioRxiv a license to display the preprint in perpetuity. It is made available under a CC-BY 4.0 International license.

Figure S1. Stepwise human B cell differentiation generates PB/prePC and PC populations *in vitro*. (A) Upregulation of PB-associated CD38 and CD27 was monitored by flow cytometry at D4 & D7. Representative plots are shown for indicated timepoints. (B, C) PB and PC differentiation were monitored by surface immunophenotyping. (B) Representative flow cytometry plots show gating strategies used to quantify actBs ($CD20^+CD38^+CD27^-$), PBs ($CD20^-CD38^+CD27^+ICAM2^{+/-}CD138^-$), and PCs ($CD20^-CD38^+CD27^+ICAM2^+CD138^+$) at D7 and D21. (C) Bar graphs show PB and PC frequencies among live cells at the indicated timepoints (Donor 2). Dots indicate independent experiments, and error bars indicate mean +/- SD (n=4-6; *p<0.05, **p<0.01; two-way ANOVA with Tukey's post-hoc test). (D) Histograms comparing CD20 staining and gating frequencies in surface-flow (Fig. 1B and S1B) and intracellular-staining analyses (Fig. 1D, E and S1E, F) at the indicated timepoints. (E, F) Intracellular flow cytometry was used to measure BLIMP1, IRF4, IRF8, and Ki-67. Cells were gated as $CD20^+$ (blue) or $CD20^-$ (orange) to relate intracellular measurements of the indicated TFs to the surface marker-defined PB/PC trajectory. (E) Biplots showing BLIMP1 and IRF4 expression at D7 (left) and D21 (right). (F) Histograms normalized to modal counts are shown for IRF8 and Ki-67 at D7 (dashed) and D21 (solid).

bioRxiv preprint doi: https://doi.org/10.64898/2026.06.15.732353; this version posted June 19, 2026. The copyright holder for this preprint (which was not certified by peer review) is the author/funder, who has granted bioRxiv a license to display the preprint in perpetuity. It is made available under a CC-BY 4.0 International license.

Fig. S2

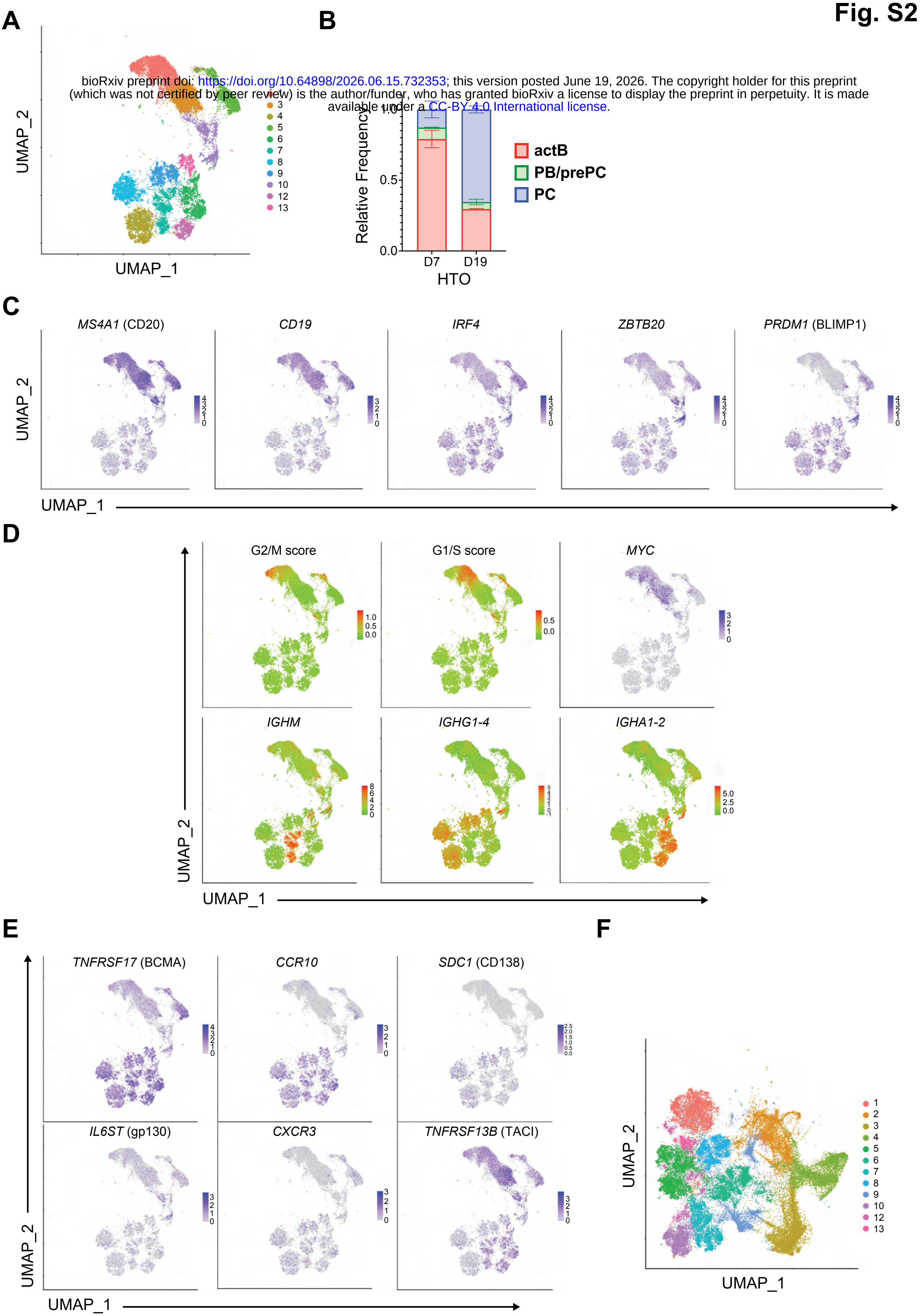

bioRxiv preprint doi: https://doi.org/10.64898/2026.06.15.732353; this version posted June 19, 2026. The copyright holder for this preprint (which was not certified by peer review) is the author/funder, who has granted bioRxiv a license to display the preprint in perpetuity. It is made available under a CC-BY 4.0 International license.

Figure S2. Single-cell transcriptional profiling resolves PB/prePC, PC, and isotype-associated states. (A) UMAP displays aggregated cells across timepoints (D7 and D19) and donors (Donors 1 and 2), annotated by Leiden cluster. (B) Bar plots show the relative frequency of each cell state at the indicated timepoints (actB, p=0.0013; PC, p=0.0011; two-way ANOVA with Sidak's multiple-comparison test). (C) UMAP projections show normalized expression of selected genes associated with PB/PC differentiation. (D) UMAP projections show cell-cycle module scores and *MYC* expression (top) and isotype scores derived from immunoglobulin heavy-chain transcript levels (bottom). (E) UMAP projections show normalized expression of selected genes associated with PC homing and survival. (F) UMAP projection shows CCA-like integration of *in vitro*-derived cells and human BMPCs annotated by Leiden cluster.

Fig. S3

bioRxiv preprint doi: https://doi.org/10.64898/2026.06.15.732353; this version posted June 19, 2026. The copyright holder for this preprint (which was not certified by peer review) is the author/funder, who has granted bioRxiv a license to display the preprint in perpetuity. It is made available under a CC-BY 4.0 International license.

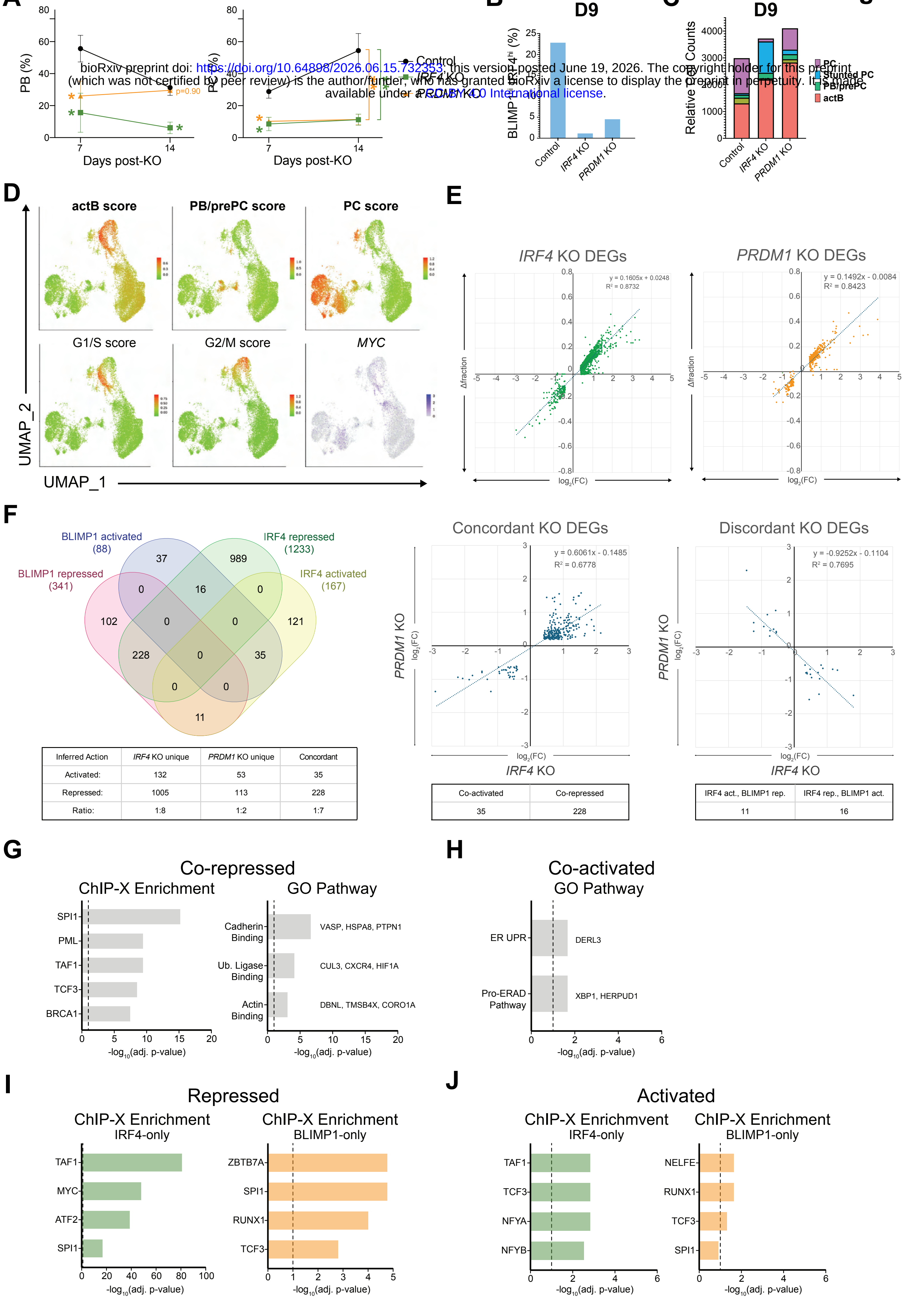

Inferred Action	*IRF4* KO unique	*PRDM1* KO unique	Concordant
Activated:	132	53	35
Repressed:	1005	113	228
Ratio:	1:8	1:2	1:7

Co-activated	Co-repressed
35	228

IRF4 act., BLIMP1 rep.	IRF4 rep., BLIMP1 act.
11	16

bioRxiv preprint doi: https://doi.org/10.64898/2026.06.15.732353; this version posted June 19, 2026. The copyright holder for this preprint (which was not certified by peer review) is the author/funder, who has granted bioRxiv a license to display the preprint in perpetuity. It is made available under a CC-BY 4.0 International license.

Figure S3. Stage-specific perturbations reveal distinct functions of IRF4 and BLIMP1 in prePCs. (A) Effects of stage-specific *IRF4* and *PRDM1* perturbations on PC differentiation were analyzed by immunophenotyping. Bar plots show mean PB and PC frequencies among live cells at D14 and D21 for Donor 2 (n=2 independent experiments; $^{**}p<0.01$, $^{***}p<0.001$; two-way ANOVA with Dunnett's post-hoc test for KO versus Control). (B) Effects of *IRF4* and *PRDM1* perturbation on IRF4 and BLIMP1 protein abundance were analyzed by intracellular flow cytometry. Bar plots show BLIMP1^{hi}IRF4^{hi} frequencies at D9 for Donor 2, related to Fig. 3B. (C) scRNA-seq analysis of Control, *IRF4* KO, and *PRDM1* KO cells at D9. Bar plots show the distribution of cluster cell numbers across samples at D9 for Donor 2, related to Fig. 3C. (D) UMAP projections show cell-state module scores derived from Fig. 2A, cell-cycle scores, and *MYC* expression. (E) Correlation between $\log_2$(fold-change) and the change in fraction of cells expressing each DEG after *IRF4* or *PRDM1* KO relative to the indicated reference groups (*IRF4* KO: Stunted PCs versus PCs; *PRDM1* KO PCs versus Control PCs, see Methods). Amplitudes are shown as $\log_2$(fold-change) [KO – control], and frequencies are shown as the difference in fraction of expressing cells [KO – control]. (F) Venn diagram shows intersections among IRF4 and BLIMP1 DEGs, with unique and concordant DEG numbers summarized in the table below (left). Scatter plots show $\log_2$(fold-change) relationships for concordant (R^2=0.68) and discordant (R^2=0.77) KO DEG sets (right). (G, H) Bar plots show ChIP-X and/or gene ontology pathway enrichments for genes co-repressed (G) or co-activated (H) by IRF4 and BLIMP1, with representative genes indicated. (I, J) Bar plots show ChIP-X enrichment of target gene sets for IRF4-only or BLIMP1-only repressed (I) and activated genes (J).

bioRxiv preprint doi: https://doi.org/10.64898/2026.06.15.732353; this version posted June 19, 2026. The copyright holder for this preprint (which was not certified by peer review) is the author/funder, who has granted bioRxiv a license to display the preprint in perpetuity. It is made available under a CC-BY 4.0 International license.

Fig. S4

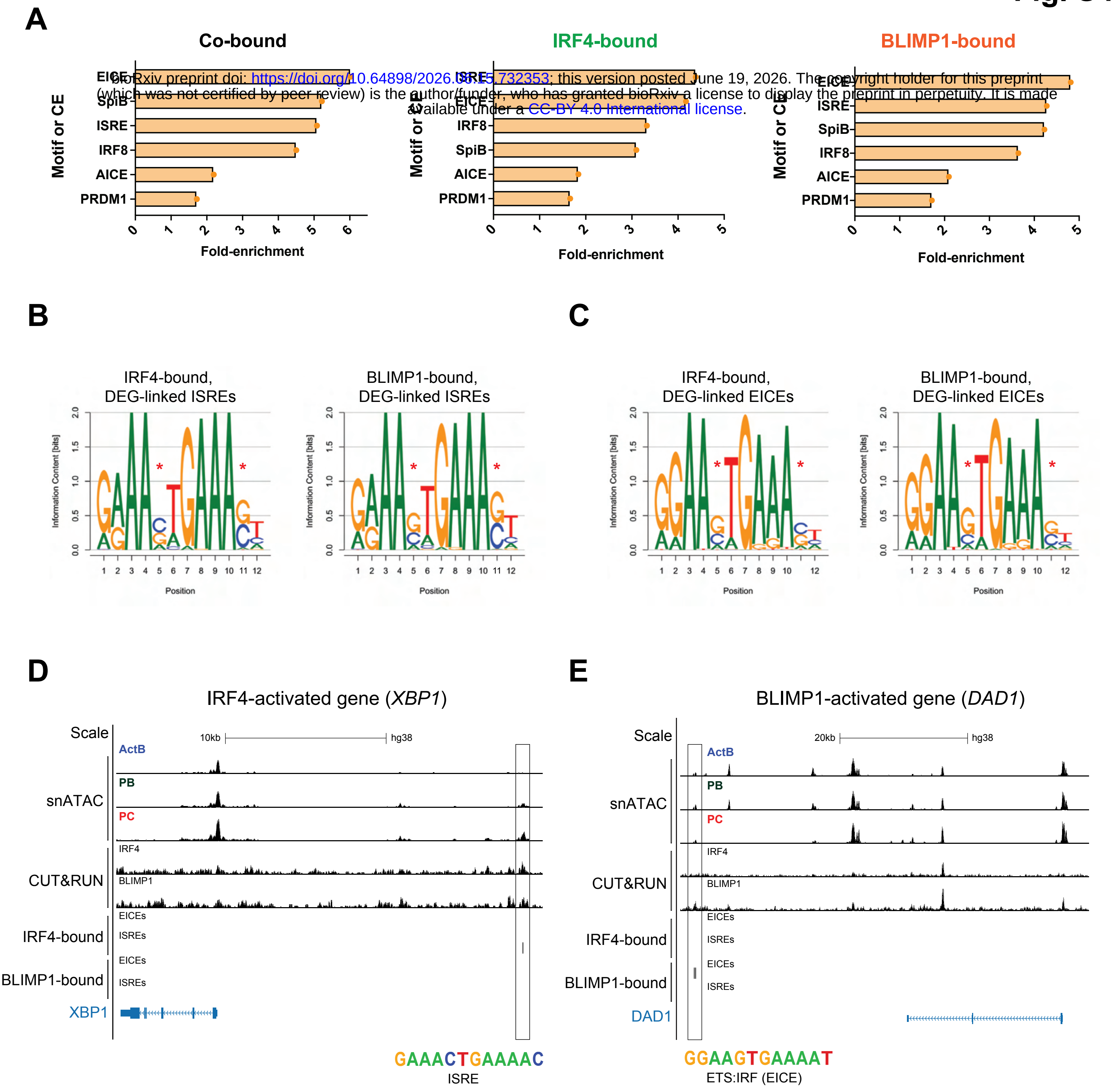

bioRxiv preprint doi: https://doi.org/10.64898/2026.06.15.732353; this version posted June 19, 2026. The copyright holder for this preprint (which was not certified by peer review) is the author/funder, who has granted bioRxiv a license to display the preprint in perpetuity. It is made available under a CC-BY 4.0 International license.

Figure S4. Differential IRF4 and BLIMP1 genomic occupancy reveals a discriminating ISRE/EICE motif lexicon. (A) Bar plots show motif enrichment analysis for selected IRF-related motifs in co-bound, IRF4-bound, and BLIMP1-bound OCRs. (B, C) Sequence logos show regenerated PWMs for (B) ISRE and (C) EICE motif instances at IRF4-bound OCRs linked to *IRF4* KO DEGs or BLIMP1-bound OCRs linked to *PRDM1* KO DEGs, related to Fig. 4D and 4E. (D) UCSC Genome Browser tracks show an example of an IRF4-bound ISRE linked to the IRF4-activated gene *XBP1*. (E) UCSC Genome Browser tracks show an example of a BLIMP1-bound EICE linked to the BLIMP1-activated gene *DAD1*.

bioRxiv preprint doi: https://doi.org/10.64898/2026.06.15.732353; this version posted June 19, 2026. The copyright holder for this preprint (which was not certified by peer review) is the author/funder, who has granted bioRxiv a license to display the preprint in perpetuity. It is made available under a CC-BY 4.0 International license.

Fig. S5

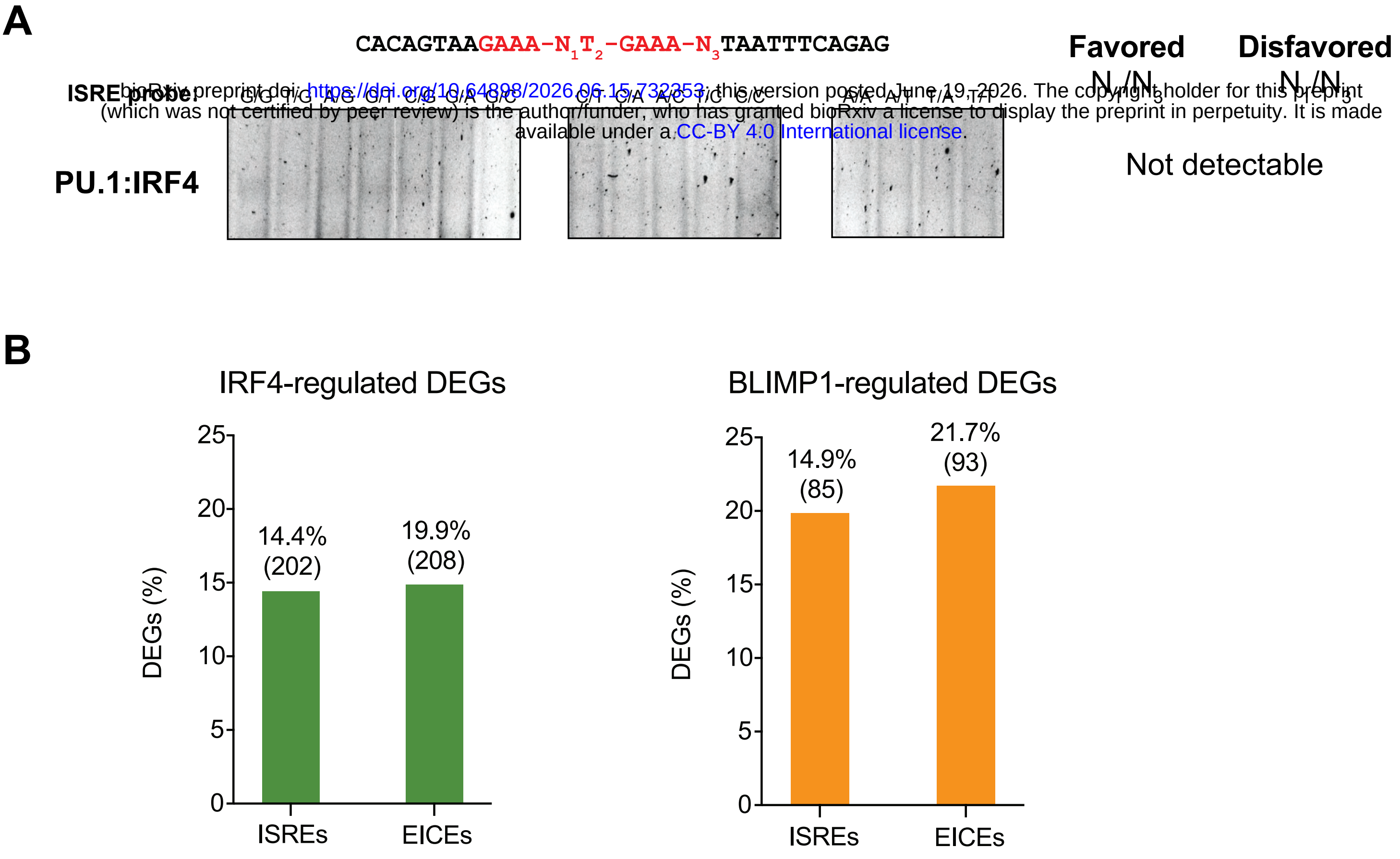

bioRxiv preprint doi: https://doi.org/10.64898/2026.06.15.732353; this version posted June 19, 2026. The copyright holder for this preprint (which was not certified by peer review) is the author/funder, who has granted bioRxiv a license to display the preprint in perpetuity. It is made available under a CC-BY 4.0 International license.

Figure S5. ***In vitro*** **TF binding assays test base-pair rules and substantiate the ISRE/EICE regulatory logic.** (A) EMSA analysis of IRF4 binding to ISRE probes in the presence of PU.1 instead of Ab directed against IRF4 epitope tag, related to Fig. 5B. (B) Comparison of ISRE and EICE association with IRF4 and BLIMP1 DEG-linked OCRs. Bar plots show the frequency of IRF4 (left) or BLIMP1 (right) DEGs linked to IRF4-bound or BLIMP1-bound OCRs containing ISRE or EICE motifs. The number of DEGs in each category is shown in parentheses above the bar plots.
